# Ageing and latent CMV infection impact on maturation, differentiation and exhaustion profiles of T-cell receptor gammadelta T-cells

**DOI:** 10.1038/s41598-017-05849-1

**Published:** 2017-07-14

**Authors:** Martine J. Kallemeijn, Anne Mieke H. Boots, Michèle Y. van der Klift, Elisabeth Brouwer, Wayel H. Abdulahad, Jan A. N. Verhaar, Jacques J. M. van Dongen, Anton W. Langerak

**Affiliations:** 1 Department of Immunology, Laboratory for Medical Immunology, Erasmus MC, Rotterdam, 3000 CA The Netherlands; 2 Department of Rheumatology, University Medical Centre Groningen, Groningen, 9700 RB The Netherlands; 3 Department of Orthopedics, Erasmus MC, Rotterdam, 3000 CA The Netherlands

## Abstract

Ageing is a broad cellular process, largely affecting the immune system, especially T-lymphocytes. Additionally to immunosenescence alone, cytomegalovirus (CMV) infection is thought to have major impacts on T-cell subset composition and exhaustion. These impacts have been studied extensively in TCRαβ+ T-cells, with reduction in naive, increase in effector (memory) subsets and shifts in CD4/CD8-ratios, in conjunction with morbidity and mortality in elderly. Effects of both ageing and CMV on the TCRγδ+ T-cell compartment remain largely elusive. In the current study we investigated Vγ- and Vδ-usage, maturation, differentiation and exhaustion marker profiles of both CD4 and CD8 double-negative (DN) and CD8+TCRγδ+ T-cells in 157 individuals, age range 20–95. We observed a progressive decrease in absolute numbers of total TCRγδ+ T-cells in blood, affecting the predominant Vγ9/Vδ2 population. Aged TCRγδ+ T-cells appeared to shift from naive to more (late-stage) effector phenotypes, which appeared more prominent in case of persistent CMV infections. In addition, we found effects of both ageing and CMV on the absolute counts of exhausted TCRγδ+ T-cells. Collectively, our data show a clear impact of ageing and CMV persistence on DN and CD8+TCRγδ+ T-cells, similar to what has been reported in CD8+TCRαβ+ T-cells, indicating that they undergo similar ageing processes.

## Introduction

Ageing is a general cellular process, defined as the result of damage created by reactive oxygen species (ROS) during oxidative stress in mitochondria^1^. ROS can cause cell membrane, protein, nucleic acid damage^2^, and most importantly genome damage which leads to genomic instability, shortening of telomere length, and thus an increasing chance of cancer development^3, 4^. The process of ageing particularly affects the immune system, due to its high metabolic rate and high cellular turnover for maintaining homeostasis, and for protecting the host against infections and cancer. Immunological ageing (also called immunosenescence) is defined at different levels: desensitization of dendritic cells (DCs) leading to reduced TLR responses, low bone marrow (BM) output of naive B-cells, insufficient T-cell help in the spleen and lymph nodes (LN), resulting in decreased memory B-cell expansions and antibody secretion, and decreased thymopoiesis in the thymus^5, 6^. Clinically this results in an inadequate response to infections in elderly, caused by reduced innate responses of macrophages, neutrophils and NK-cells^6, 7^. DCs are constitutively activated, which gives rise to an increased basal level of inflammation with increased tissue damage^8, 9^. Defective antigen presentation and a reduced B-cell repertoire lead to a reduced humoral response^10^ and a reduced vaccine response^6, 11^.

A central feature in immunosenescence is involution of the thymus, which is characterized by thymic shrinkage and a significantly reduced naive T-cell output^5, 6, 12^. This leads to a reduced T-cell dependent antigen-specific response and thus fewer interactions with other immune cell types, such as reduced help to B-cells in germinal centers^11^. During immune ageing, another major event has been described, which is referred to as T-cell exhaustion. This exhaustion process is characterized by the progressive loss of robust effector functions and eventually the induction of apoptosis. T-cell exhaustion is most clearly seen in chronic infections, e.g. in persistent viral infections, and in cancers. Due to continuous stimulation, T-cells start to lose their effector functions in a hierarchical manner, starting with reduced IL-2 production, followed by reduced cytokine and chemokine productions, ending with the high expression of inhibitory molecules and eventually the induction of apoptosis^13, 14^. Many different markers for exhausted CD8+ CTLs have been described, ranging from NK-cell markers such as CD57^15–17^, killer cell lectin-like receptor G1 (KLRG1)^13, 18, 19^, and 2B4, also known as CD244^20–22^, to cell death-associated markers such as Programmed cell death 1 (PD1) which is a marker of early exhaustion^23–25^, and FAS (CD95)^13, 26^. Loss of the self-renewal-associated marker IL-7 receptor α subunit (CD127) is associated with an early stage of exhaustion^27, 28^.

The process of immunological ageing, including expression of the above markers has been extensively studied in TCRαβ+CD8+ T-cells, but less so in TCRγδ+ T-cells, which show functional overlap with the TCRαβ+CD8+ CTLs with respect to high levels of cytotoxicity^29^, cytokine release – mainly IFN-γ and IL-17 based on antigen experience^30, 31^, induction of inflammation, immunoregulation and cytoprotection upon antigen recognition. However, TCRγδ+ T-cells form a distinctive group of unconventional T-cells with features of both innate and adaptive immune cells^32^. TCRγδ+ T-cells recognize antigens directly without major histocompatibility molecules (MHC), or in the context of CD1-molecules^33–35^. TCRγδ+ T-cells thus have the ability to directly respond to specific pathogens, and readily form a bridge between the innate and adaptive systems. Upon ageing, TCRγδ+ T-cells also tend to decrease in total numbers^36, 37^, leading to a possibly reduced response to pathogens. This relates not only to the blood, but also to epithelial tissues where they reside as intra-epithelial and innate-like lymphocytes^33, 38^. Furthermore, TCRγδ+ T-cells can specifically bind to viruses, such as human lymphotrophic virus type I (HTLV-I) and Epstein-Barr virus (EBV)^39^, through the Vγ9/Vδ2 receptor. Non-Vγ9/Vδ1 cells specifically respond to cytomegalovirus (CMV)^40^, increase upon ageing and can be expanded and stimulated with CMV *ex vivo* ^41^. As CMV is one of the persistent herpesviruses^42^, CMV infection has a high impact on immunosenescence and exhaustion^43–45^. CMV is known for altering TCRαβ+CD4+ and CD8+ maturation subsets (reviewed in ref. 46), and recently it has been found that CMV seropositivity in elderly individuals is associated with a lower percentage of Vδ2+, and an increased percentage of Vδ1+ TCRγδ+ T-cells, of which the latter has a late-stage differentiated effector phenotype^37^. However, the full profile of phenotypic alterations of TCRγδ+ T-cells upon ageing in the presence or absence of persistent CMV infections remains elusive.

Since TCRγδ+ T-cells have innate features and show functional similarities to TCRαβ+CD8+ CTLs, we hypothesized that immunological ageing, especially in the presence of CMV, would similarly influence the TCRγδ+ T-cell immune system with respect to subset compositions and exhaustion profiles. In the current study we included 157 healthy subjects from different age groups to investigate the effect of both ageing and CMV seropositivity on TCRγδ+ T-cells. Our data illustrate the impact of immunological ageing on TCRγδ+ T-cells, with a clear enhancing effect of CMV, as opposed to the more marginal contribution of CMV infection to increased TCRγδ+ T-cell exhaustion in elderly.

## Results

### Clear decline in absolute numbers of the most common Vγ9/Vδ2 TCRγδ+ T-cell subset in peripheral blood of elderly subjects

**Figure 1:**
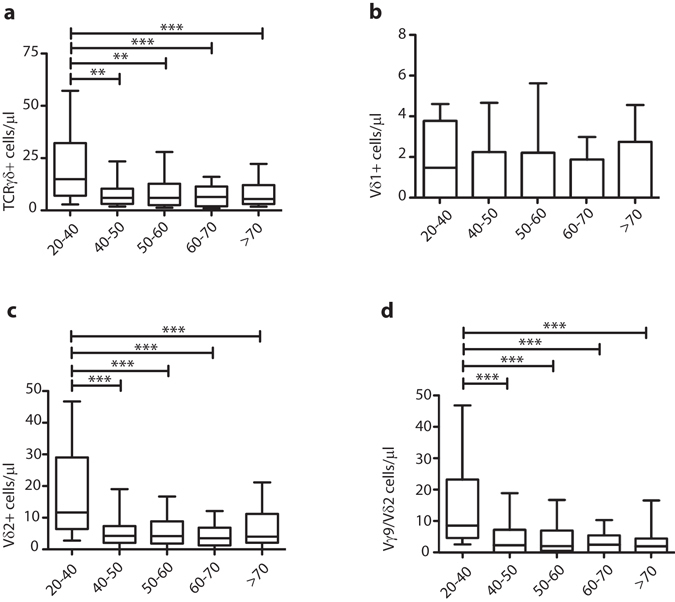
Vγ/Vδ-gene usage in TCRγδ+ T-cells in different age groups. (**a**) Absolute numbers of total TCRγδ+ T-cells, (**b**) Vδ1+, (**c**) Vδ2+ and (**d**) Vγ9/Vδ2 TCRγδ+ T-cells depicted as 10–90% box-whisker-plots. Significance was tested by a Kruskal-Wallis test, followed by a post-Dunn’s test to correct for multiple testing. Significance for the Dunn’s test is indicated in the plots: **p < 0.01; ***p < 0.001.

When studying absolute numbers of TCRγδ expressing T-cells, a significant decrease was observed with ageing, which was already apparent at age group 40–50 (Fig. 1a). In contrast, absolute numbers of TCRαβ+ T-cells were hardly or not affected, although variation was high in elderly (Supplementary Fig. 2a). As a consequence the overall distribution of TCRαβ versus TCRγδ expressing T-cells showed a significant increase in TCRαβ+ T-cell frequencies and a significant decrease in TCRγδ+ T-cell frequencies, again already at age group 40–50 (Supplementary Fig. 2b). When focusing more on subsets with specific Vδ receptor usage no significant differences in the absolute numbers of Vδ1+ cells were found (Fig. 1b). However the significant decrease in total TCRγδ+ T-cells was rather paralleled by a significant decrease in Vδ2+ cells (Fig. 1c), and especially Vγ9/Vδ2 cell populations (Fig. 1d). To determine whether this resulted in a clear shift in Vδ usage within the total TCRγδ+ T-cell population, we then compared the distributions of Vδ1, Vδ2 and other Vδ (non-Vδ1, non-Vδ2) populations in the peripheral blood of all age groups. We found no significant alterations, although the percentage of Vδ2+ cells was decreased in age groups 40–50, 50–60 and 60–70, with a shift towards relatively more Vδ1+ cells (Supplementary Fig. 2c). Overall these data suggest a significant decrease in absolute numbers of TCRγδ+ T-cells in elderly age groups, with a parallel decrease in numbers of the most dominant Vγ9/Vδ2 TCRγδ+ T-cell population.

### During ageing the naive TCRγδ+ T-cell compartment shrinks and shifts towards a late-differentiated effector phenotype

**Figure 2:**
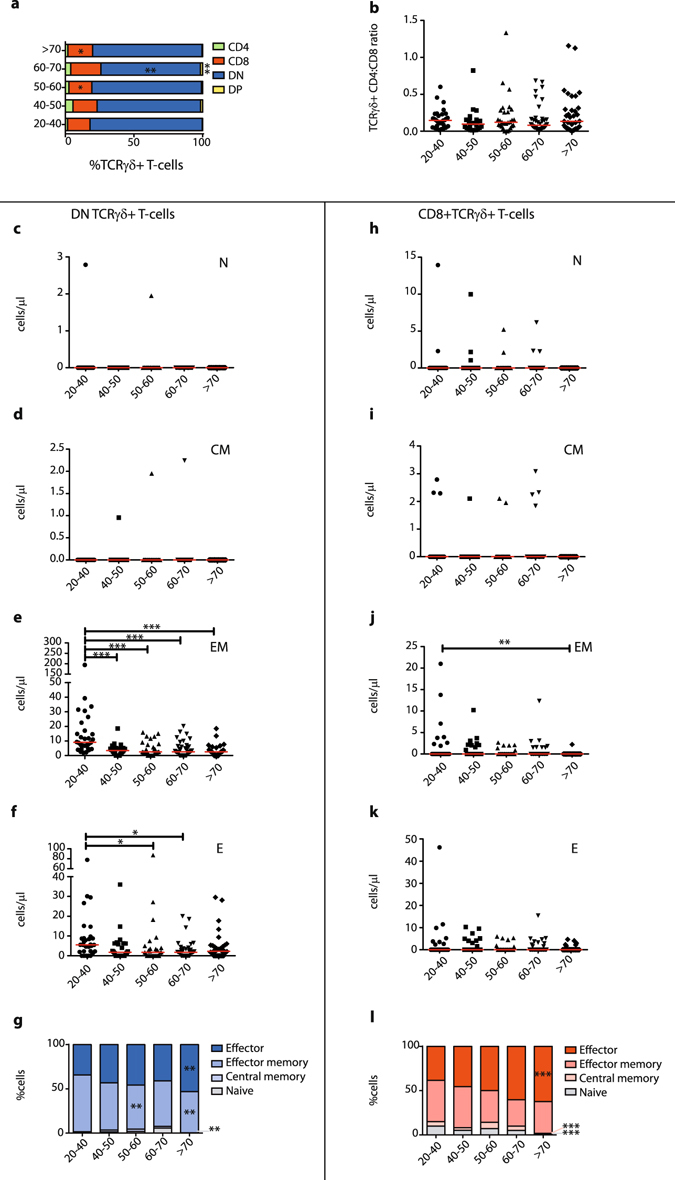
TCRγδ+ T-cell maturation statuses and subset distributions during ageing. (**a**) Relative visualization of CD4 and CD8 single-positive, double-positive (DP, CD4+CD8+) and double-negative (DN, CD4−CD8−) distribution within the total TCRγδ+ T-cell compartment between different age groups. (**b**) CD4:CD8 ratios in total TCRγδ+ T-cell population. (**c**) Absolute numbers of DN naive (CD45RO−CD197+), (**d**) central memory (CD45RO+CD197+), (**e**) effector memory (CD45RO+CD197−, Temro) and (**f**) effector (CD45RO−CD197−, Temra) TCRγδ+ T-cells. (**g**) Relative distributions of maturation subsets of DN TCRγδ+ T-cells depicted in stacked bar plots. (**h**) Absolute numbers of CD8+ naive, (**i**) central memory, (**j**) effector memory and (**k**) effector CD8+TCRγδ+ T-cells. (**l**) Relative distributions of maturation subsets of CD8+TCRγδ+ T-cells depicted in stacked bar plots. Ratios and absolute numbers are indicated in scatter plots indicated with the median. Significance was tested by a Kruskal-Wallis test, followed by a post-Dunn’s test for correction for multiple testing. Significance for the Dunn’s test is indicated in the plots: *p < 0.05; **p < 0.01, ***p < 0.001.

In analogy to the effects described for CD8+ cytotoxic T-lymphocytes (CTL), we next investigated maturation and differentiation of TCRγδ+ T-cells. From age 50 onwards slight shifts in the distribution of double negative (DN), CD4 single-positive (SP), CD8 SP, and double-positive (DP) cells were visible, mostly affecting the predominant DN and CD8 SP compartments (Fig. 2a). TCRαβ+ T-cells also showed significant differences in the CD4/CD8 distributions upon ageing (Supplementary Fig. 2d), with a clear shift in the CD4/CD8 ratio towards more CD4+ T-cells (Supplementary Fig. 2e), as described before^47–50^. Even though in TCRγδ+ T-cells CD4/CD8 ratios have a completely different meaning, given that TCRγδ+ T-cells usually do not express CD4 and the CD8α dimer upon activation^38^, we still checked these ratios and did not observe significant changes (Fig. 2b).

Of note, earlier documented changes in CD8+TCRαβ+ CTL maturation were also observed in our cohort, with significant decreases in the naive and significant increases in effector CD8+TCRαβ+ T-cell compartments (Supplementary Fig. 2f), whilst CD4+TCRαβ+ T-cells did not show similar significant changes in these maturation subsets (Supplementary Fig. 2g). These TCRαβ+ T-cell results thus validate our dataset as being representative for investigating immunological ageing of TCRγδ+ T-cells. Therefore, next we determined maturation subsets of the DN and CD8+TCRγδ+ T-cells. In general, for the DN TCRγδ+ T-cell population no significant differences in absolute numbers of naive (CD45RO−CD197+) (Fig. 2c) or central memory (CD45RO+CD197+) cells (Fig. 2d) were found. In contrast, in the effector memory (CD45RO+CD197−) (Fig. 2e) and effector (CD45RO−CD197−) populations (Fig. 2f) numbers decreased significantly, with effector memory cell numbers decreasing already in the age group 40–50 and effector cell numbers decreasing mainly in age groups 50–60 and 60–70. For CD8+TCRγδ+ T-cells, only in the effector memory population a significant difference in absolute numbers was observed (Fig. 2h–k). Notably, when further studying relative distributions of these maturation subsets, which in addition to the cell numbers could reflect biologically relevant shifts in subset composition, significant differences were observed for both DN and CD8+TCRγδ+ T-cells. These concerned decreases in the naive subset fractions and increases in the effector subset fractions (Fig. 2g,l). This was especially true in the oldest age group (>70), although decreasing (naive) and increasing (effector) trends were in fact already visible from age 50 onwards.

**Figure 3:**
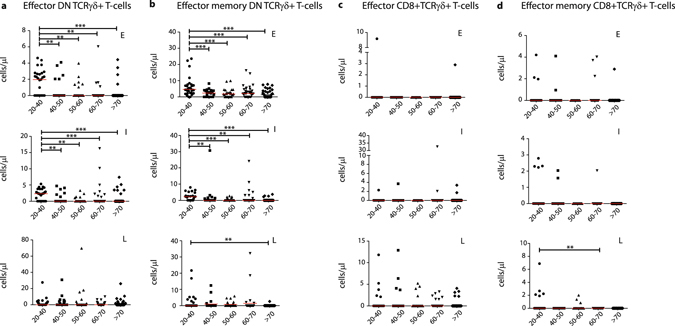
DN and CD8+TCRγδ+ effector and effector memory differentiation stages during ageing. (**a**) Absolute numbers of early (CD27+CD28+), intermediate (CD27+CD28−) and late (CD27−CD28−) differentiated DN and (**c**) CD8+TCRγδ+ effector (CD45RO−CD197−, Temra) T-cells. (**b**) Absolute numbers of early, intermediate and late differentiated DN and (**d**) CD8+TCRγδ+ effector memory (CD45RO+CD197−, Temro) T-cells. Early (E), intermediate (I) and late (L) definitions are indicated in the upper right corners of the graphs. Scatter plots are indicated with the median. Significance was tested by a Kruskal-Wallis test, followed by a post-Dunn’s test for correction for multiple testing. Significance for the Dunn’s test is indicated in the plots: **p < 0.01; ***p < 0.001.

As TCRγδ+ effector T-cells are known to have a rather late-stage differentiated phenotype^37^, we then further focused on early (CD27+CD28+), intermediate (CD27+CD28−) and late (CD27−CD28−) subpopulations. This analysis showed significant decreases in absolute numbers of early and intermediate DN TCRγδ+ effector cells starting from age group 40–50, but not of late-stage differentiated effector cells (Fig. 3a). Given that also the effector memory cells were found to relatively expand in the aged groups (Fig. 2g,l), we additionally looked into early, intermediate and late differentiated cells within the effector memory subset. Significant decreases in early and intermediate DN TCRγδ+ effector memory cell numbers were found when all age groups were compared with the 20–40 age control group. Also a significant decrease in the absolute numbers of late-stage differentiated cells was observed, mainly when the oldest age group was compared with the control group (Fig. 3b). CD8+TCRγδ+ cells showed no significant differences in early, intermediate or late stages, except for a decrease in absolute numbers of late-differentiated effector memory cells in the oldest age group (Fig. 3c,d).

**Figure 4:**
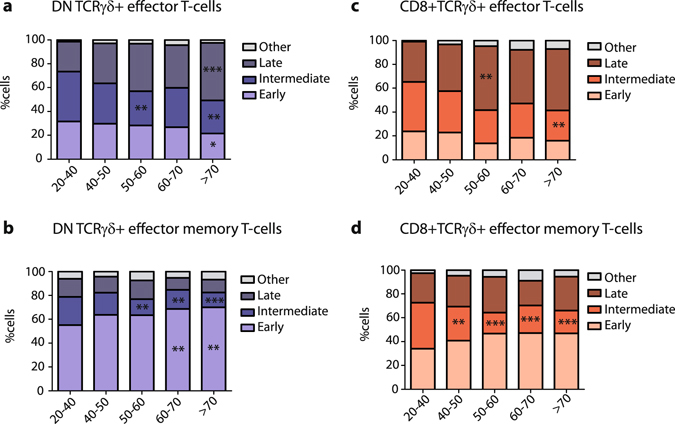
DN and CD8+TCRγδ+ effector and effector memory differentiation stage distributions during ageing. (**a**) Relative differentiated subset distributions of percentages of DN and (**c**) CD8+ effector and (**b**) DN and (**d**) CD8+ effector memory TCRγδ+ T-cells depicted in stacked bar plots. Significance was tested by a Kruskal-Wallis test, and followed by a post-Dunn’s test for correction for multiple testing. Data of different age groups was compared with the control age group. Significance for the Dunn’s test is indicated in the plots: *p < 0.05; **p < 0.01; ***p < 0.001.

Since absolute numbers of total TCRγδ+ T-cells generally decreased (Fig. 1a), and the maturation subsets displayed clear shifts in distribution (Fig. 2g,l), we also investigated the relative shifts of stages within the effector and effector memory cell populations as discussed above. Significant decreases were observed in early and intermediate DN TCRγδ+ effector cell proportions with a concomitant significant increase in percentages of late-stage differentiated cells in especially the > 70 age group when all age groups were compared to the control age group (Fig. 4a); this was rather reversed in the effector memory cells where an increase in the proportions of early differentiated cells was observed (Fig. 4b). Furthermore, even though in CD8+TCRγδ+ effector T-cells no significant changes in absolute numbers were observed (Fig. 3c), their relative distributions did show significant decreases in especially intermediate effector cells, concurrent with a significant increase in late-stage differentiated cells (Fig. 4c) at age 50–60. Within the CD8+TCRγδ+ effector memory population a significant decrease in the proportions of intermediate differentiated cells was observed (Fig. 4d).

Taken together, we conclude that ageing has a similar effect on TCRγδ+ maturation subsets as was reported for CD8+ TCRαβ+ CTL. Despite an overall decrease in TCRγδ+ cell numbers, the relative increase in effector cells and the shift towards a late-stage differentiated phenotype result in stable numbers of the most differentiated effector cell population.

### CMV seropositivity impacts on Vδ usage at old age

Persistent viruses and especially CMV are known to have major effects on the composition, senescence, and exhaustion of the immune system^13, 43, 45, 46, 51^. We therefore studied the potential impact of CMV on immunological ageing of TCRγδ+ T-cells. To this end we subdivided our study cohort, according to CMV serology. Furthermore, we also looked at gender as a potential confounding factor for immunological ageing. Although the proportion of CMV seropositive individuals was higher with age in both males and females, these percentages were not significantly different in any age group (Supplementary Fig. 3); in fact the CMV seroprevalence of our age groups correlated well with previous reports^52, 53^. Recently it was shown that gender and additionally CMV infection were associated with an expansion of late- stage differentiated TCRαβ+ T-cell subsets and a reduction of naive, regulatory and CD8+ T-cells in especially middle-aged (age category 50–65) males^54^. As we did not observe a gender effect (Supplementary Fig. 4) or specific differences in gender and CMV serology in the middle-aged 50–65 group (data not shown) with respect to TCRγδ+ T-cells in our cohort, we further focused our analyses on age and CMV serology only. In order to be able to make clear distinctions, we defined groups of young controls (age 20–40) and elderly individuals (above age 60) (Table 1), and subdivided both groups into seronegative (CMV−) and seropositive (CMV+) subjects.

**Table 1:** Age group characteristics of study subjects.

	Controls (20–40)	40–50	50–60	60–70	>70
Total number	N = 30	N = 24	N = 29	N = 40	N = 34
Age (mean ± SD)	25.3 (4.2)	45.2 (2.6)	55.1 (2.6)	65.8 (2.5)	76.9 (5.5)
Range	20–40	40–49	51–59	61–69	70–95
Males (n; %)	11 (36.7%)	10 (41.7%)	11 (37.9%)	14 (35%)	12 (40%)
CMV− (n; %)	6 (54.5%)	8 (80%)	7 (63.6%)	7 (50.0%)	4 (33.3%)
CMV+ (n; %)	5 (45.5%)	2 (20%)	4 (36.3%)	7 (50.0%)	8 (66.7%)
Females (n; %)	19 (63.3%)	14 (58.3%)	18 (62.1%)	26 (65%)	22 (60%)
CMV− (n; %)	15 (78.9%)	8 (57.1%)	10 (55.6%)	9 (34.6%)	7 (31.8%)
CMV+ (n; %)	4 (21.1%)	6 (42.9%)	8 (44.2%)	17 (65.4%)	15 (68.2%)

Values are means (SD) and absolute numbers (percentages). Percentages of CMV negative and positive individuals are from total males or females.

**Figure 5:**
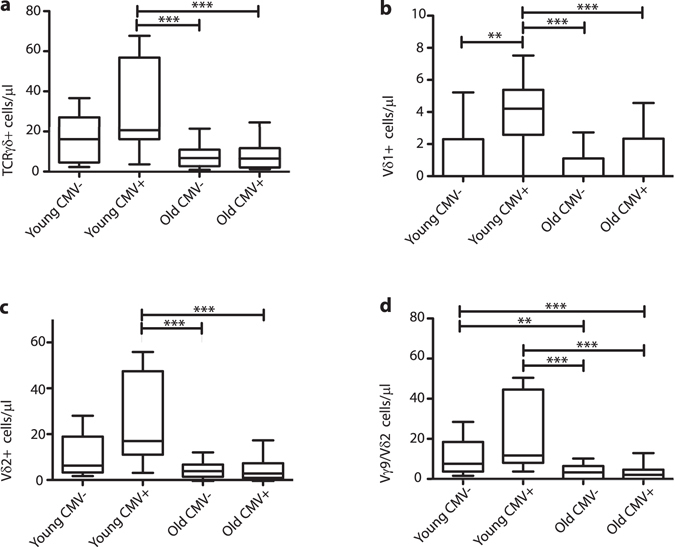
Effect of CMV seropositivity on Vγ/Vδ-usage. (**a**) Absolute numbers of total TCRγδ+ T-cells, (**b**) Vδ1+, (**d**) Vδ2+ and (**d**) Vγ9/Vδ2 TCRγδ+ T-cells depicted as 10–90% box-whisker-plots. Significance was tested by a Kruskal-Wallis test, followed by a post-Dunn’s test to correct for multiple testing. Significance for the Dunn’s test is indicated in the plots: **p < 0.01; ***p < 0.001.

First, total TCRγδ+ T-cell absolute counts were compared between young vs. elderly, and CMV− vs. CMV+ groups, which showed a significantly higher total TCRγδ+ T-cell count in young CMV+ individuals (Fig. 5a). In elderly the absolute numbers of total TCRγδ+ T-cells were decreased, without a significant additional effect of CMV (Fig. 5a). Of note, TCRαβ+ T-cell counts were not significantly affected in these subgroups (Supplementary Fig. 2h). The relative distributions of TCRαβ+ and TCRγδ+ T-cells were also determined, showing significant alterations in elderly; particularly in CMV+ elderly the percentage of TCRγδ+ T-cells was significantly reduced, with a parallel increase of the TCRαβ+ T-cell fraction (Supplementary Fig. 2i). Furthermore, upon evaluation of Vγ/Vδ-usage, changes in absolute numbers of Vδ2+ and Vγ9/Vδ2 populations were largely similar as for total TCRγδ+ T-cells, whilst absolute numbers of Vδ1+ cells were increased in both young and old CMV+ individuals (Fig. 5c,d). The overall Vδ-usage distribution showed significant changes in the composition in especially CMV-infected elderly, with an increased proportion of Vδ1+ and a decreased proportion of Vδ2+ cells (Supplementary Fig. 2j). The increase in CMV seroprevalence in elderly (Supplementary Fig. 3) and the relative increase in Vδ1 usage (Supplementary Fig. 2j) in both elderly and young CMV+ individuals correlate with known Vδ1+ cell reactivity to CMV^55^.

Collectively, these data suggest that CMV has a profound effect on the numbers of all TCRγδ+ T-cells in young infected individuals, and that in elderly the impact of CMV is more distinct TCRγδ+ subgroups showing different Vδ usage.

### The shift towards an effector TCRγδ+ T-cell phenotype in elderly is largely explained by CMV infection

**Figure 6:**
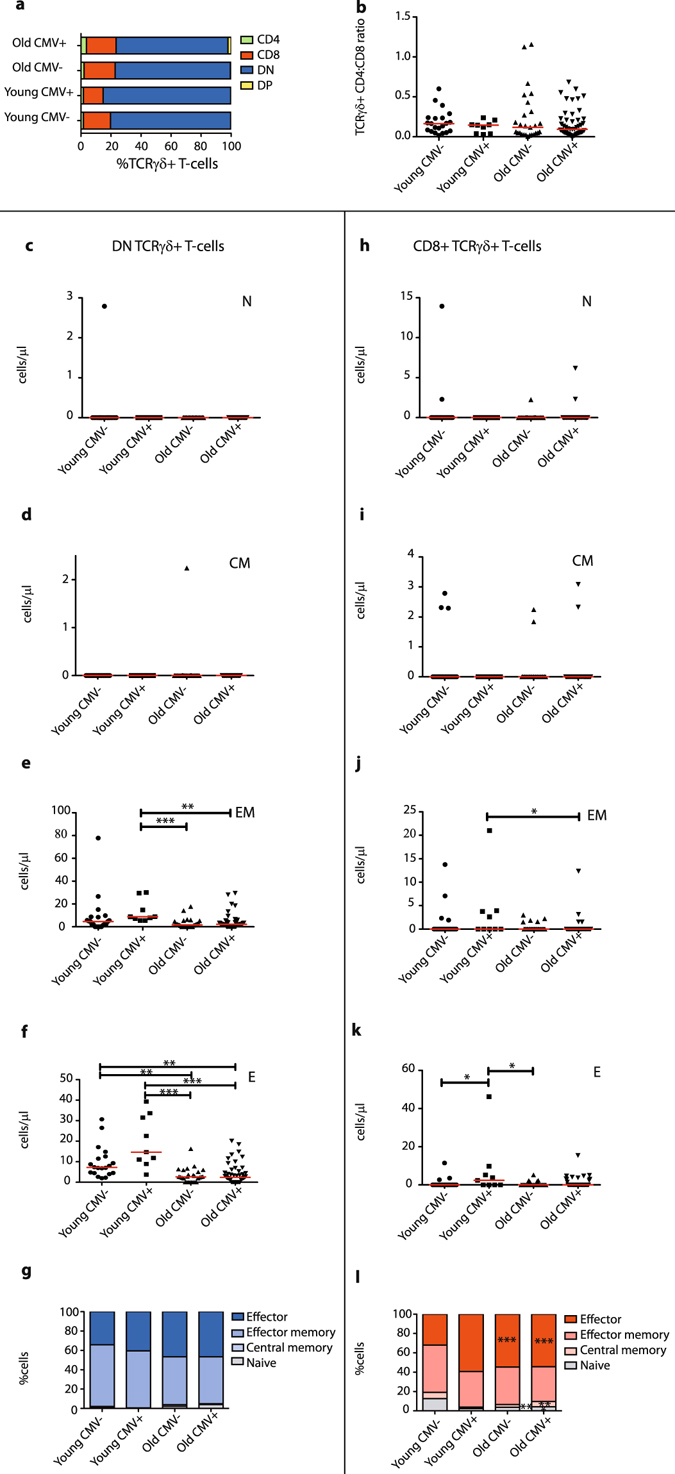
Effect of CMV on TCRγδ+ T-cell maturation. (**a**) Relative visualization of CD4 and CD8 single-positive, double-positive (DP, CD4+ CD8+) and double-negative (DN, CD4−CD8−) distribution within the total TCRγδ+ T-cell compartment between different age and CMV groups. (**b**) CD4:CD8 ratios in total TCRγδ+ T-cell population. (**c**) Absolute numbers of DN naive (CD45RO−CD197+), (**d**) central memory (CD45RO+CD197+), (**e**) effector memory (CD45RO+CD197−, Temro) and (**f**) effector (CD45RO−CD197−, Temra) TCRγδ+ T-cells. (**g**) Relative distributions of maturation subsets of DN TCRγδ+ T-cells depicted in stacked bar plots. (**h**) Absolute numbers of CD8+ naive, (**i**) central memory, (**j**) effector memory and (**k**) effector CD8+ TCRγδ+ T-cells. (**l**) Relative distributions of maturation subsets of CD8+ TCRγδ+ T-cells depicted in stacked bar plots. Ratios and absolute numbers are indicated in scatter plots indicated with the median. Significance was tested by a Kruskal-Wallis test, followed by a post-Dunn’s test for correction for multiple testing. Significance for the Dunn’s test is indicated in the plots: *p < 0.05; **p < 0.01, ***p < 0.001.

Next we also investigated the impact of CMV on maturation phenotypes of TCRγδ+ T-cells. Firstly, no significant alterations were seen in the frequencies of the predominant DN and CD8+ TCRγδ+ T-cell populations (Fig. 6a) or in the CD4/CD8 ratios of TCRγδ+ T-cells (Fig. 6b), both in the absence and presence of CMV. In contrast, CD4, CD8, DN, DP populations in TCRαβ+ T-cells were significantly different, mainly in the elderly CMV- group (Supplementary Fig. 2k), with a shift in the CD4/CD8 ratio towards more CD4+ T-cells (Supplementary Fig. 2l). In keeping with published data, clear changes were seen in the relative proportions of different maturation stages in the CD4+ and CD8+ TCRαβ+ T-cells upon CMV (Supplementary Fig. 2m,n), thus reinforcing the validity of our cohort for studying TCRγδ+ T-cells.

When focusing on the absolute numbers of naive and central memory DN TCRγδ+ cells no significant changes were observed (Fig. 6c,d). In contrast, we did find significant changes in effector memory and effector cell absolute counts, with increased numbers being present in especially the young CMV+ group (Fig. 6e,f). In the CD8+TCRγδ+ T-cell population we did not observe significant changes in the naive and central memory subsets either (Fig. 6h,i), whilst again the young CMV+ group showed higher effector memory and effector cell counts (Fig. 6j,k) in keeping with the significant increase in total TCRγδ+ T-cells in young CMV+ individuals (Fig. 5a). In order to further investigate the biological impact of both ageing and CMV we then also focused on the relative subset distributions. We did not find significant differences in the subset distribution of DN TCRγδ+ T-cells, despite an increasing trend in the effector population (Fig. 6g). However, the relative distributions of CD8+TCRγδ+ T-cell maturation subsets did significantly alter. Especially upon the presence of CMV, the percentages of effector cells were increased, with a concomitant decrease in the naive compartment (Fig. 6l). The subset distribution pattern of young CMV+ individuals reflected that of elderly (Fig. 6l).

**Figure 7:**
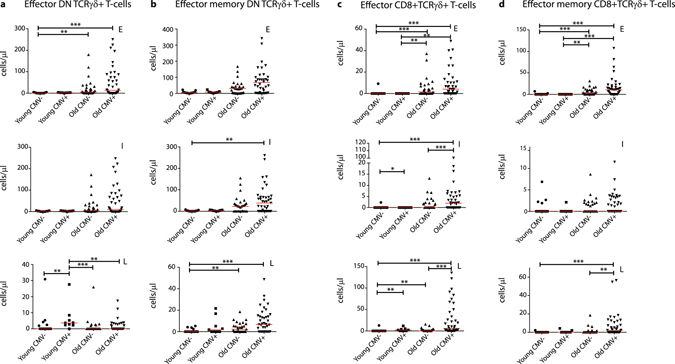
Effect of CMV on effector and effector memory differentiation stages. (**a**) Absolute numbers of early (CD27+CD28+), intermediate (CD27+CD28−) and late (CD27−CD28−) differentiated DN and (**c**) CD8+TCRγδ+ effector (CD45RO−CD197−, Temra) T-cells. (**b**) Absolute numbers of early, intermediate and late differentiated DN and (**d**) CD8+TCRγδ+ effector memory (CD45RO+CD197−, Temro) T-cells. Early (E), intermediate (I) and late (L) definitions are indicated in the upper right corners of the graphs. Scatter plots are indicated with the median. Significance was tested by a Kruskal-Wallis test, followed by a post-Dunn’s test for correction for multiple testing. Significance for the Dunn’s test is indicated in the plots: **p < 0.01; ***p < 0.001.

When looking more in-depth into the differentiation stages within effector and effector memory cells, the absolute numbers of early and intermediate effector DN TCRγδ+ T-cells increased, whilst late effector cells showed a significant increase in predominantly the young CMV+ individuals (Fig. 7a). The effector memory DN TCRγδ+ T-cells showed increased numbers in all differentiation stages when it comes to ageing in the presence of CMV, although this was not significant for the early differentiated cells (Fig. 7b). In CMV+ elderly the absolute numbers of CD8+TCRγδ+ effector and effector memory cells were significantly higher in almost all differentiation stages, except for intermediate effector memory cells (Fig. 7c,d).

**Figure 8:**
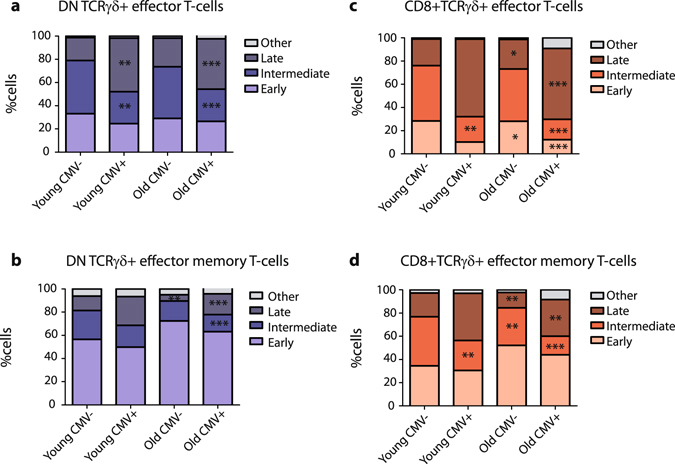
DN and CD8+TCRγδ+ effector and effector memory differentiation stage distributions during ageing and in absence or presence of CMV. (**a**) Relative differentiated subset distributions of percentages of DN and (**c**) CD8+ effector and (**b**) DN and (**d**) CD8+ effector memory TCRγδ+ T-cells depicted in stacked bar plots. Significant differences between all groups was tested by a Kruskal-Wallis test, and followed by a post-Dunn’s test for correction for multiple testing. Significance for the Dunn’s test is indicated in the plots: *p < 0.05; **p < 0.01; ***p < 0.001.

As we observed in all differentiation stages an increase in absolute numbers in elderly CMV+ individuals, we then further looked into the relative composition. When evaluating the overall distribution patterns, comparing all groups with each other, young and old CMV− individuals were showing very similar distribution patterns, as well as young and old CMV+ individuals (Fig. 8). In the presence of CMV − in both young and elderly – percentages of DN TCRγδ+ intermediate effector cells decreased, whilst those of late effector cells increased (Fig. 8a). This effect was similar and even more pronounced for the CD8+TCRγδ+ effector population (Fig. 8c). When investigating effector memory subpopulations, analogous to what was seen in elderly age groups, significant increases in absolute numbers and relative distributions were noted, mainly in CMV+ elderly (Fig. 8b,d).

Altogether these data clearly indicate that the presence of CMV greatly impacts on the absolute numbers of differentiated effector and effector memory populations, as well as induces shifts in maturation subset compositions of TCRγδ+ T-cells similar to what is seen in elderly, i.e. a main shift towards effector and effector memory phenotypes, showing more late-staged differentiated effector cells and early-stage differentiated effector memory cells.

### CMV infection marginally contributes to the increased exhaustion profile of TCRγδ+ T-cells in elderly

**Figure 9:**
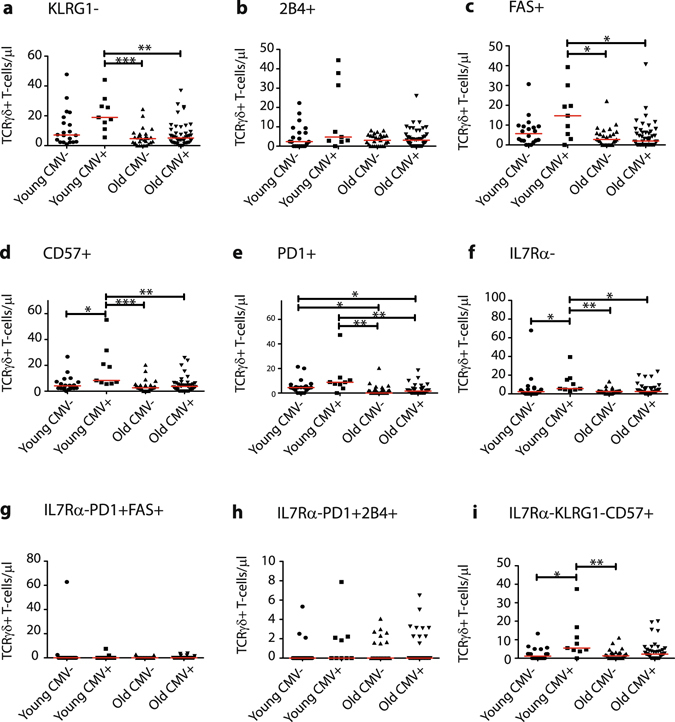
Effect of CMV on exhaustion and senescence profiles of TCRγδ+ T-cells. (**a**) Absolute numbers of TCRγδ+ T-cells lacking KLRG1 expression, (**b**) TCRγδ+ T-cells expressing 2B4, (**c**) FAS death receptor, (**d**) CD57, (**e**) PD1, (**f**) TCRγδ+ T-cells lacking IL7Rα, (**g**) and IL7Rα− TCRγδ+ T-cells co-expressing PD1 and FAS, (**h**) PD1 and 2B4, and (**i**) expressing CD57 and lacking KLRG1. Scatter plots are indicated with the median. Significance was tested by a Kruskal-Wallis test, and followed by a post-Dunn’s test for correction for multiple testing. Significance for the Dunn’s test is indicated in the plots: *p < 0.05; **p < 0.01; ***p < 0.001.

T-cell exhaustion is a phenomenon that is often seen in persistent viral infections like CMV^13, 14, 56^, and that largely shapes the CD8+TCRαβ+ T-cell compartment of the immune system^42, 46, 51^. Within our cohort we also observed increased absolute numbers (Supplementary Fig. 5) and percentages (Supplementary Fig. 6) of CD8+ non-TCRγδ (TCRαβ) T-cells expressing exhaustion markers. As the level of exhaustion of TCRγδ+ T-cells during ageing and upon the presence of persistent viral infections like CMV has not been properly documented, we investigated absolute numbers of total TCRγδ+ T-cells expressing or lacking the senescence and exhaustion associated markers. We observed increased absolute numbers of KLRG1− (Fig. 9a), FAS+ (Fig. 9b), CD57+ (Fig. 9d), PD1+ (Fig. 9e) and IL7Rα− (Fig. 9f) TCRγδ+ T-cells especially in the context of CMV, in both young and elderly. 2B4+ TCRγδ+ T-cells were also increased in case of young CMV+ individuals, although this was not significant (Fig. 9b). Since senescence and exhaustion processes are difficult to separate, and since there are no concrete definitions, we also looked into combinations of different markers. IL7Rα is lost already during early stages of both exhaustion and senescence, and therefore we studied the marker combinations within the IL7Rα− TCRγδ+ T-cell population (Fig. 9g–i). We observed only a significant increase in IL7Rα−KLRG1−CD57+ TCRγδ+ T-cells, mainly in the young CMV+ population (Fig. 9i).

In view of our findings of increased TCRγδ+ T-cell numbers in young but not old CMV+ individuals (Fig. 5a), we considered an exhaustion phenotype in especially elderly and thus looked for the fractions of TCRγδ+ T-cells showing exhaustion markers. When analyzing the percentages increasing trends could be appreciated, however there were no significant differences observed, although the variation among younger individuals was higher when compared to elderly, independent of CMV infection (Supplementary Fig. 7).

Overall, these data suggest that immunological ageing does contribute to a more increased exhaustion phenotype of TCRγδ+ T-cells, and that CMV plays an additional role.

## Discussion

With increasing hygiene and improved health care individuals in the Western world become significantly older according to the World Health Organization^57, 58^. Immunological ageing, also referred to as immunosenescence, has large impacts on elderly individual’s health as evidenced from less efficient responses to infectious agents^11^, reduced vaccine responses^5, 6^, and increased risks of developing cancer due to insufficient anti-tumor activity of the immune system^59^. Immunosenescence has been described at different levels in the immune system with the most well-defined effect of ageing concerning subset changes in TCRαβ+CD4 + and CD8+ T-cells^47–50^. T-cell exhaustion, characterized by a stepwise loss of effector functions, plays a major role in immunosenescence^13^. This has mainly been investigated in the TCRαβ+ T-cell compartment as well, most notably affecting CD8+ T-cells. We hypothesized that ageing has similar effects on the TCRγδ+ T-cell population, which could largely affect the elderly individuals due to the reduced or aberrant response of TCRγδ+ T-cells to pathogens. TCRγδ+ T-cells implement different functions from both innate and adaptive immunity by readily responding to antigens in both CD8+ CTL and NK-cell like manners^33^. The majority of our data presented here is based on absolute counts, in order to determine direct effects, but in some cases this is complemented with information about biological shifts in the overall composition of TCRγδ+ T-cells. We conclude from our data that ageing decreases absolute numbers of total TCRγδ+ T-cells, without significantly affecting the absolute numbers of TCRαβ+ T-cells, starting at age 40–50, confirming earlier findings^60^. This decrease in total TCRγδ+ T-cells mostly affected the most common TCRγδ+ T-cell type in the peripheral blood: Vγ9/Vδ2 cells^61^. In contrast, Vδ1+ cells showed a slight increasing trend in absolute numbers during ageing, implicating a potential role of Vδ1+ -specific antigens which could maintain this population over time. Immunological ageing is not solely defined by chronological ageing, but also by persistent viruses like CMV^56^. Vδ1+ cells are often CMV specific^41, 55, 62^, and CMV+ individuals have higher numbers of Vδ1+ cells, as described in earlier studies^40, 45^. This could explain the increasing trend in absolute numbers and percentages of Vδ1+ cells as observed in our cohort in elderly individuals. When CMV serology was included in the analysis, we observed a significant increase in absolute counts of total TCRγδ+ T-cells in young CMV+ individuals, in line with previous findings^37^. This could indicate a prolonged activation state, even during early phases of latency, as described by van de Berg *et al*.^63^.

This latency of e.g. CMV does not only influence cells bearing specific receptors for its epitopes, it also shapes the immune system in terms of maturation subsets. Ageing alone caused a significant decrease in total TCRγδ+ T-cells, and thus decreasing absolute numbers of effector and effector memory phenotype cells. However, when considering relative TCRγδ+ T-cell subset distributions a significant increase in effector and effector memory phenotypes were observed, similar to what has been described for TCRαβ+CD8+ T-cell subset distributions^64^, suggesting similar underlying ageing processes. The effect of ageing became more evident when CMV serological status was included in the analysis, showing increased absolute numbers of effector and effector memory cells. Again, when evaluating relative subset distributions, the effects of both ageing and CMV persistence became more evident, for both maturation and effector and effector memory differentiation stages. We mainly found decreased proportions of naive, and increased proportions of effector cells, which have a late-stage differentiated profile, as described before^37, 46^. In contrast to the effector cells, we observed increased proportions of early-stage differentiated effector memory cells, which could correlate with a more general memory-like response to antigens in elderly upon prior antigen exposure. Furthermore, we could observe an age-independent effect of CMV on the differentiation stages of effector and effector memory TCRγδ+ T cells, which correlates with previous data of Roux *et al*.^45^.

Immune ageing is however not only accompanied by shifts from naive to effector cells, since effector cells also progressively lose their function (exhaustion) during the ageing process^13^. We noticed a significantly enlarged TCRγδ+ T-cell population expressing exhaustion-related markers in young CMV+ individuals, while CMV serology did not add to T-cell exhaustion in elderly. Loss of IL7Rα marked the loss of self-renewal and early stages of T-cell exhaustion. We observed a clear increase in absolute counts of IL7Rα− TCRγδ+ T-cells in elderly. Also, we observed increased expression of CD57, which is also highly expressed on terminally differentiated CD8+ CTL with high proliferative activity^15^, thus marking replicative senescence and susceptibility to activation-induced cell death (AICD)^16^. High expression is also associated with chronic viral infections like CMV^17^. Our data showed significantly increased numbers of CD57+ TCRγδ+ T cells, in both young and old CMV+ individuals. Another NK-cell marker which is associated with T-cell exhaustion is KLRG1, of which high expression marks terminally differentiated or senescent T-cells^13, 18^, whilst severely exhausted CD8+ CTLs are KLRG1-negative^19^. We observed higher numbers of KLRG1-negative cells in CMV infected individuals. Also, our data showed that exhausted CD8+ CTL cells were present in higher numbers in CMV+ individuals. Combination of loss of IL7Rα, high CD57 and low KLRG1 expression may function as a marker of exhausted cells. Our data confirmed this with an increased cell count of such exhausted cells, especially already in the young CMV+ group. Furthermore, we saw increasing but not significant numbers of TCRγδ+ T-cells expressing the NK-cell marker 2B4 (CD244), which is normally expressed on memory CD8+ T-cells^20, 21^. The percentages did show an increasing trend from young to elderly, but this was not as evident as described earlier^13^. Of note, 2B4 expression is positively associated with Programmed cell death 1 (PD1) in exhausted CD8+ T-cells^23^, mediating decreasing TCR-mediated proliferation and cytokine production by providing downstream inhibitory signals^24, 25^. Our data did show significant increases in PD1+ TCRγδ+ T-cells counts, but IL7Rα−PD1+2B4+ TCRγδ+ T-cell counts were not altered. IL7Rα− TCRγδ+ T-cells co-expressing PD1 with the apoptosis inducer FAS (CD95) did not significantly alter upon ageing and CMV persistence, however, total PD1+ TCRγδ+ T-cells did increase upon CMV persistence, although this was only most obvious for the young CMV+ individuals. However, PD1 is also associated with T-cell activation, and might be in case of young CMV+ individuals more indicative of a response to CMV, rather than exhaustion.

In order to further investigate the full exhaustion and senescence profile, and to better discriminate between these processes, it would be relevant to extend marker analysis to other exhaustion markers, such as CD160, Tim3 and Lag3 in combination with PD1 and 2B4^65^, and to assess transcription factors that define T-cell subsets (such as FoxP3, Blimp1, Eomes, T-bet)^66, 67^. Furthermore, functional analyses would be helpful to assess *in vitro* the proliferative potential, activation status and apoptosis of TCRγδ+ T-cells from CMV− and CMV+ individuals at young and old age. Also, studying the epigenetic landscape of exhaustion-, senescence- and activation-related gene profiles of TCRγδ+ T-cells could shed more insights on the actual effects of ageing on TCRγδ+ T-cells^68^.

In summary, we conclude that ageing by itself impacts on TCRγδ+ T-cells, leading to a decrease in the absolute counts of total TCRγδ+ T-cells and to shifts in maturation and differentiation subsets. Furthermore, CMV has an additional impact on TCRγδ+ T-cell receptor usage, maturation subsets, effector differentiation profiles, as recently reviewed by Khairallah *et al*.^69^, and ultimately on exhaustion marker profiles. This indicates that TCRγδ+ T-cells are subjected to ageing and exhaustion processes in much the same way as CD8+ TCRαβ+ CTLs.

## Methods

### Study subjects

The NWO ageing study cohort consisted of immunologically healthy patients from the Orthopedics outpatient clinic, Erasmus MC, complemented with (immunologically) healthy controls in the younger age groups. After applying exclusion criteria (auto-immune or –inflammatory diseases at present or in the past; malignancies; usage of anti-inflammatory or immunosuppressive drugs; surgery in the past 30 days; alcohol or drug abuse) a total of 121 subjects were included. To increase the number of subjects in especially the age groups of >60 years, an additional 36 subjects participating in the SENEX study of healthy elderly in the Dutch region of Groningen were included via the UMC Groningen. Participants in the NWO ageing study gave written informed consent and the study was approved by the Medical Ethics Committee of the Erasmus MC under number MEC-2011–409 and MEC-2016–202. From subjects participating in the SENEX Study of the UMC Groningen written informed consent was obtained and approval for the study was provided by the Medical Ethics Committee of the UMCG under protocol number 2012375. All experimental studies were conducted in accordance with relevant guidelines and principles of the Declaration of Helsinki. Samples were divided into five age groups, 40 to 50 (mean age 45), 50 to 60 (mean age 55), 60 to 70 (mean age 66), 70-plus (mean age 77) versus a control group of samples from healthy adults age 20 to 40 (mean age 25) (Table 1). Fresh peripheral blood mononuclear cells (PBMC) samples of a total of 157 participants of the NWO and SENEX studies were analyzed after lysis with ammonium chloride. CMV serostatus was determined on plasma with the use of the anti-CMV ELISA (IgG) according to the manufacturer’s protocol (EuroImmun, Lübeck, Germany). Remaining peripheral blood after analysis and plasma storage was subjected to Ficoll-Paque (density 1.077 g/ml, Pharmacia, Uppsala, Sweden) density gradient separation and cryopreserved in Iscove’s Modified Dulbecco’s Medium (IMDM, Lonza, Basel, Switzerland) with dimethyl sulfoxide in vials at −180 °C until further use.

### Flow cytometric immune phenotyping

Freshly obtained blood was lysed with ammonium chloride and washed with phosphate buffered saline (PBS) pH 7.8 containing fetal bovine serum (FBS, 30% w/v) and sodium azide. Samples were stained using three antibody panels according to Supplementary Table 1. Data analysis and gating strategies were based on the standardized protocols from the Generation R study^70^. Defining viable cells was based on FSC/SSC gating strategies and validated with negative expression of Annexin V of viable cells in a small series of additional samples (data not shown). With the use of tube 1 Vγ- and Vδ-usage could be determined. With the use of T-cell maturation markers CD45RO, CD197, CD27 and CD28 in the second tube maturation and differentiation statuses of TCRγδ+ T-cell populations could be determined: naive (CD45RO− CD197+), central memory (CD45RO+CD197+), circulating effector memory (CD45RO+CD197−, also known as Temro cells), and effector (CD45RO−CD197−, also known as Temra cells) TCRγδ+ T-cells. Furthermore, CD27 and CD28 were used for subdivision into early-, intermediate- and late-stage differentiated effector memory and effector cells. Tube 3 included markers for the evaluation of exhaustion profiles. Cells were acquired using the Fortessa LSR flow cytometer (BD Biosciences, San Jose, CA, USA). Compensation was based on single color controls. Data were analyzed with FACSDiva software (BD Biosciences). The gating strategy applied for the various tubes is displayed in Supplementary Figure S1. The absolute cell count per microliter of a particular TCRγδ+ T-cell population was calculated based on the total percentage and the total absolute lymphocyte count. The latter was calculated with the use of the number of events in the lymphocyte gate using the TruCount tube (BD Biosciences) and the Canto II flow cytometer (BD Biosciences) (Supplementary Fig. 1a), the initial white blood cell count and the number of leukocyte events.

### Statistical analysis

The non-parametric one-way ANOVA Kruskal-Wallis test was performed to compare absolute numbers and frequencies between different age groups and between young and elderly CMV− and CMV+ groups on single immune subsets. The Dunn’s test was applied for correction for multiple testing. P-values of <0.05 were considered statistically significant. The statistical analyses were performed in Prism 5 (GraphPad, La Jolla, CA, USA).

## Electronic supplementary material

Suppl. Material
