# Supplementary material for: Ageing and latent CMV infection impact on maturation, differentiation and exhaustion profiles of T-cell receptor gammadelta T-cells
Source: Sci Rep. 2017 Jul 14;7:5509. doi: 10.1038/s41598-017-05849-1 (PMC5511140; doi:10.1038/s41598-017-05849-1)
Supplement: Supplementary file 1 — Suppl. Material [file 41598_2017_5849_MOESM1_ESM.pdf]

Supplementary Information

Ageing and latent CMV infection impact maturation, differentiation and exhaustion profiles of T-cell receptor gammadelta T cells

Martine J. Kallemeijn[1], Anne Mieke H. Boots[2], Michèle Y. van der Klift[1], Elisabeth Brouwer[2], Wayel H. Abdulahad[2], Jan A.N. Verhaar[3], Jacques J.M. van Dongen[1], Anton W. Langerak[1].

Supplementary Figures

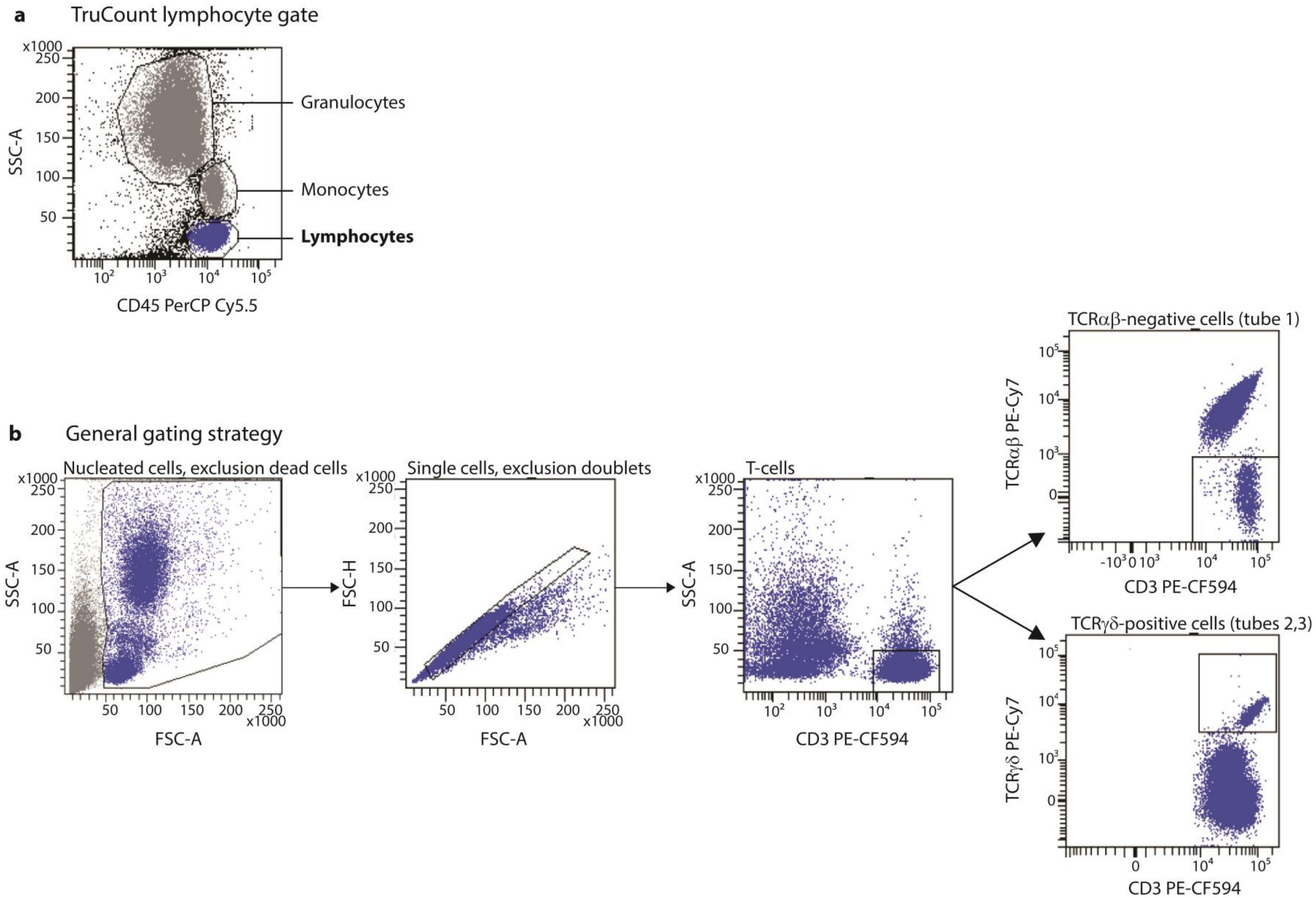

c Vγ/ Vδ gating strategy within TCRαβ-negative gate (tube 1)

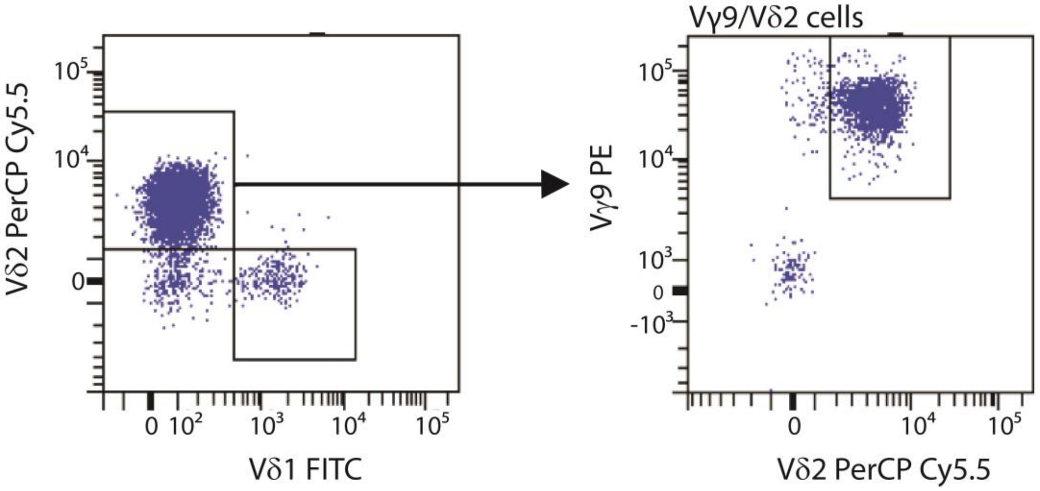

d CD4/CD8, maturation and differentiation gating strategy within TCRγδ-negative and -positive gates

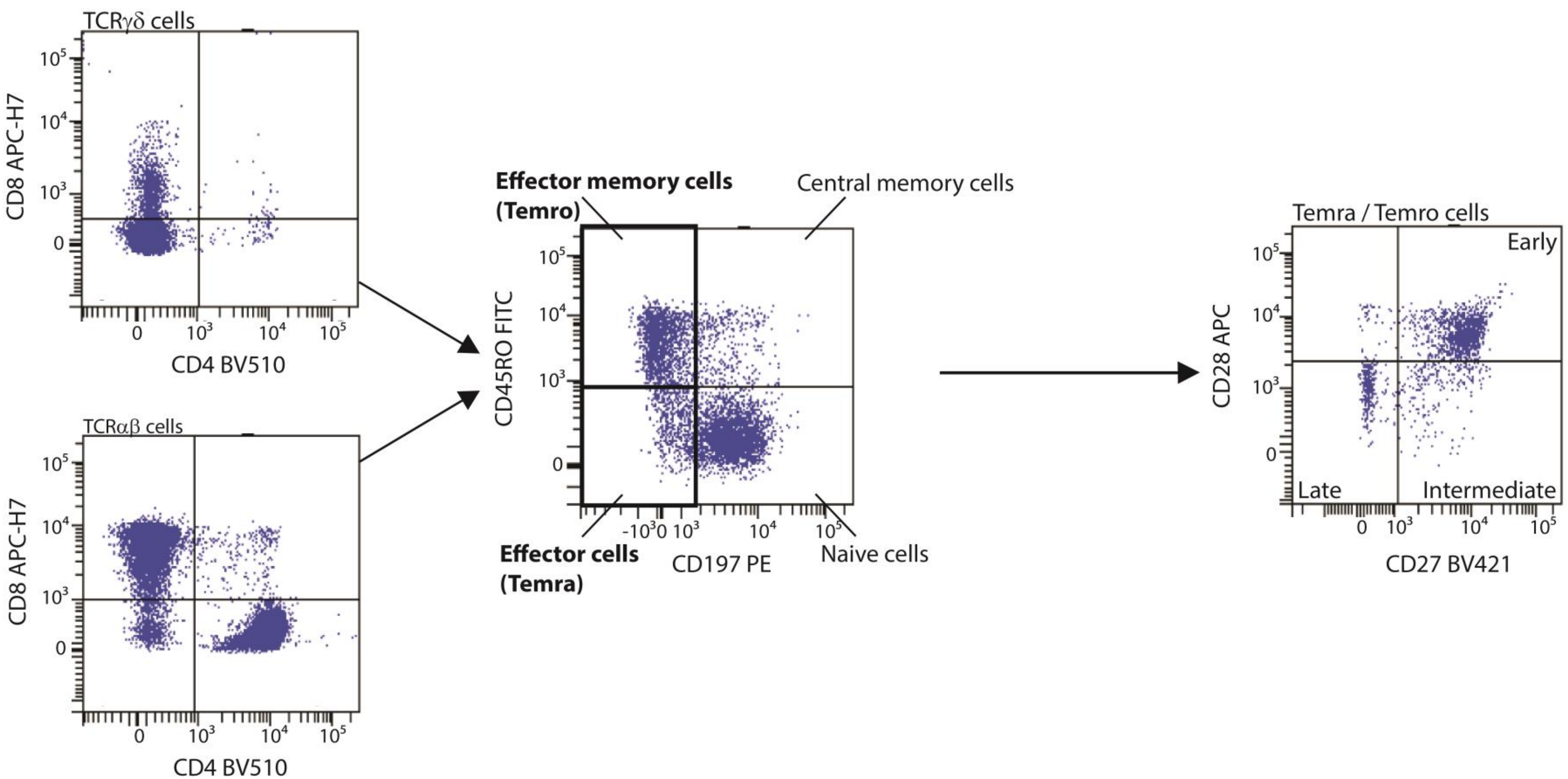

e Exhaustion gating strategy

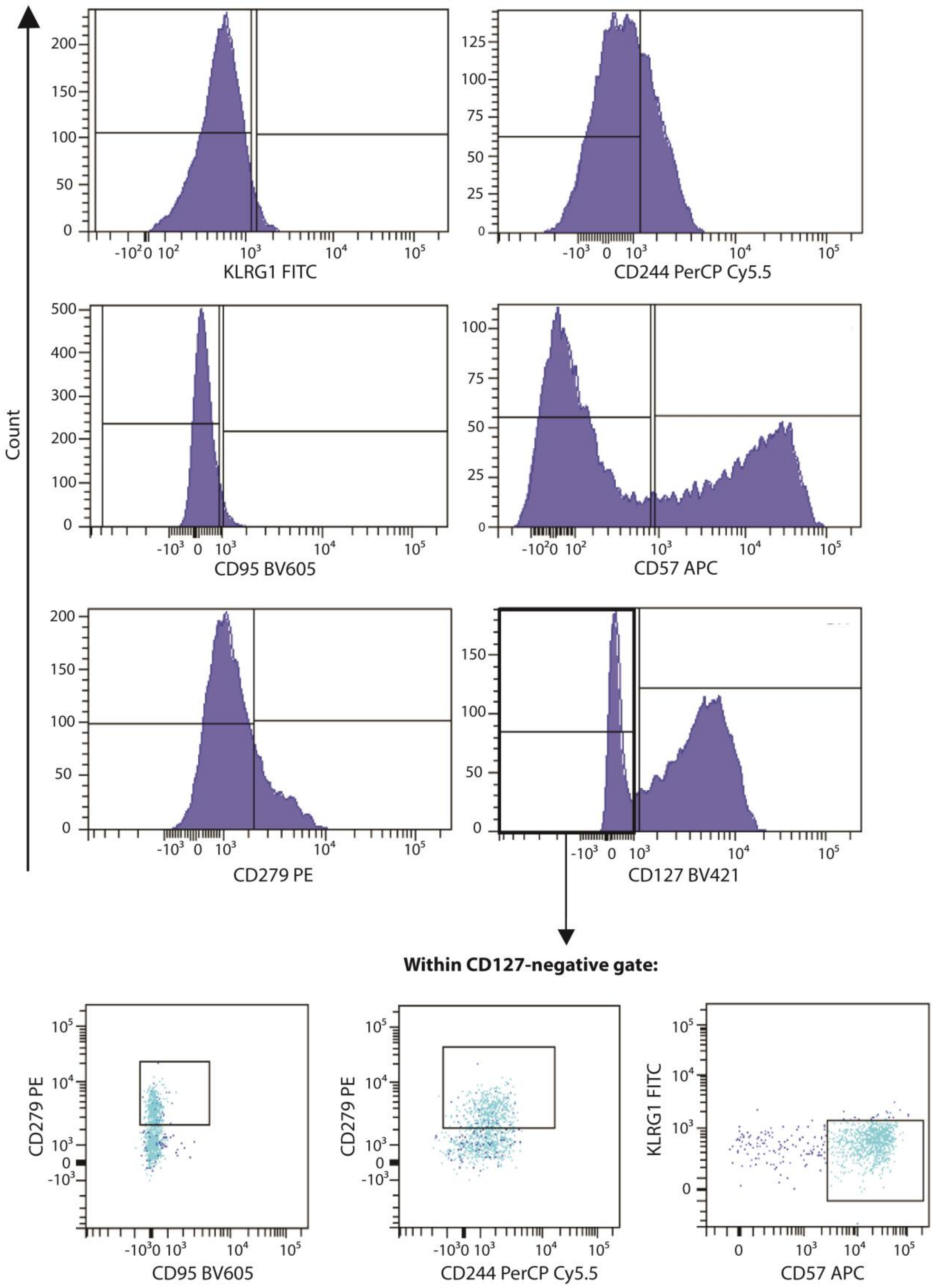

Figure S1. Gating strategies for analysis. (a) TruCount gating strategy for determining absolute lymphocyte counts and absolute cell number calculations. **(b)** General gating strategy with the use of TCRαβ antibody in case of Vγ- and Vδ-usage investigation, **(c)** gating strategy using TCRγδ antibody. **(d)** CD4/CD8 usage, maturation and differentiation within effector (Temra) and effector memory (Temro) populations. **(e)** Exhaustion profile determination including separate exhaustion marker expressions and exhaustion marker combinations within the CD127- population.

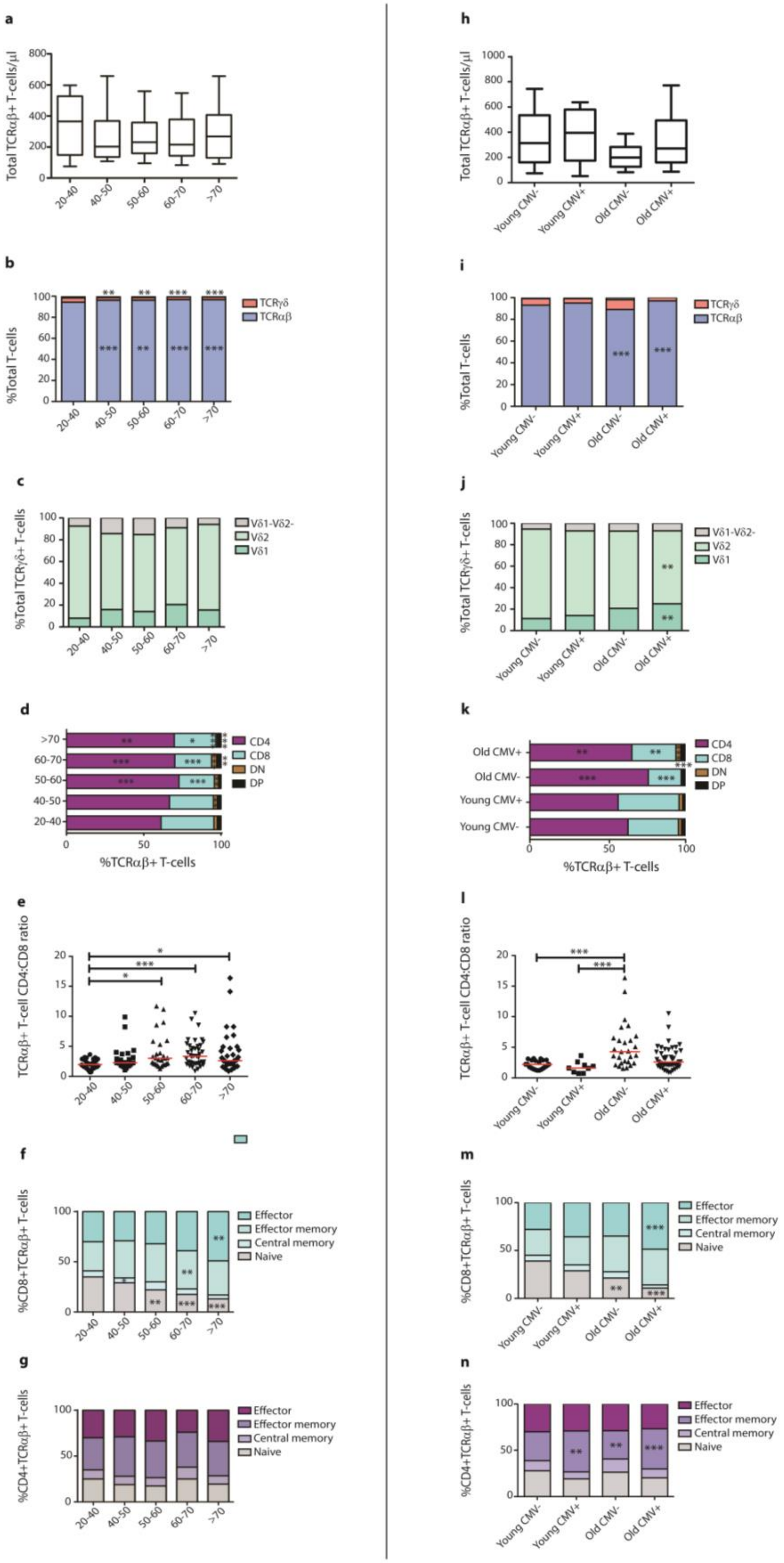
a
Total TCRαβ+ T-cells/µl
20-40
40-50
50-60
60-70
>70
b
%Total T-cells
TCRγδ
TCRαβ
c
%Total TCRγδ+ T-cells
Vδ1-Vδ2-
Vδ2
Vδ1
d
CD4
CD8
DN
DP
%TCRαβ+ T-cells
e
TCRαβ+ T-cell CD4:CD8 ratio
f
%CD8+TCRαβ+ T-cells
Effector
Effector memory
Central memory
Naive
g
%CD4+TCRαβ+ T-cells
Effector
Effector memory
Central memory
Naive
h
Total TCRαβ+ T-cells/µl
Young CMV-
Young CMV+
Old CMV-
Old CMV+
i
%Total T-cells
TCRγδ
TCRαβ
j
%Total TCRγδ+ T-cells
Vδ1-Vδ2-
Vδ2
Vδ1
k
Old CMV+
Old CMV-
Young CMV+
Young CMV-
CD4
CD8
DN
DP
%TCRαβ+ T-cells
l
TCRαβ+ T-cell CD4:CD8 ratio
m
%CD8+TCRαβ+ T-cells
Effector
Effector memory
Central memory
Naive
n
%CD4+TCRαβ+ T-cells
Effector
Effector memory
Central memory
Naive

Figure S2. TCRαβ+ and TCRγδ+ T-cell supplementary figure. (a,h) Absolute numbers of TCRαβ+ T-cells depicted in 10-90% box-whiskers-plots. **(b,i)** Relative distributions of TCRαβ+ and TCRγδ+ T-cells within the total T-cell compartment of all groups. **(c,j)** Relative Vδ1+, Vδ2+, and non-Vδ1/non-Vδ2 usage distribution in the peripheral blood total TCRγδ+ T-cell compartment. **(d,k)** TCRαβ+ CD4 and CD8 single-positive, double-positive (DP, CD4+CD8+) and double-negative (DN, CD4-CD8-) subset distribution depicted in stacked bar plots. **(e,l)** CD4:CD8 ratios of total TCRαβ+ T-cells depicted in scatter plots indicated with the median. **(f,m)** Maturation subset distributions of CD8+TCRαβ+ and **(g,n)** CD4+TCRαβ+ T-cells depicted in stacked bar plots. Significance was tested by a Kruskal-Wallis test, followed by a post-Dunn's test. Significance of the Dunn's test is indicated in the plots: *, $p<0.05$; **, $p<0.01$; ***, $p<0.001$.

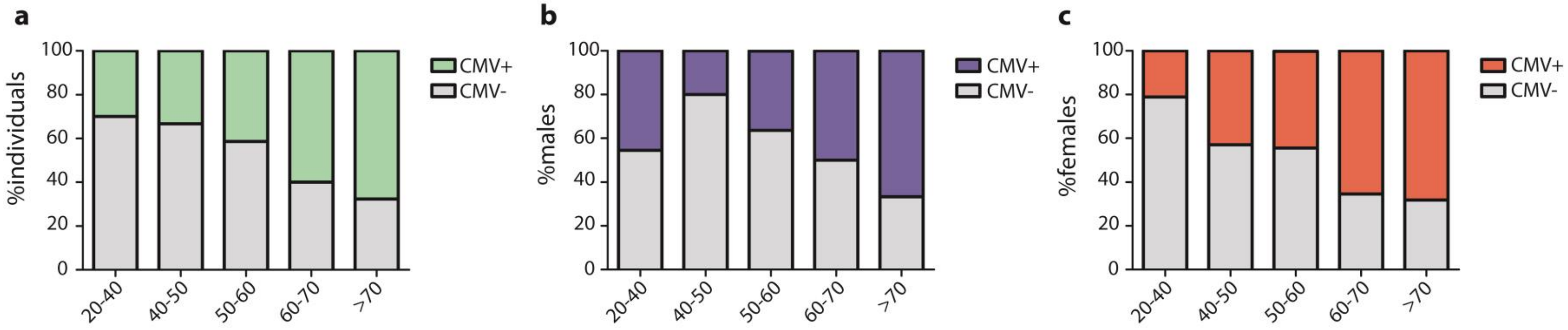

Figure S3. CMV serology. (a) Percentages of overall CMV serostatus. **(b)** CMV-positivity and –negativity among males and **(c)** females.

Significance was tested by a Kruskal-Wallis test, followed by a post-Dunn's test.

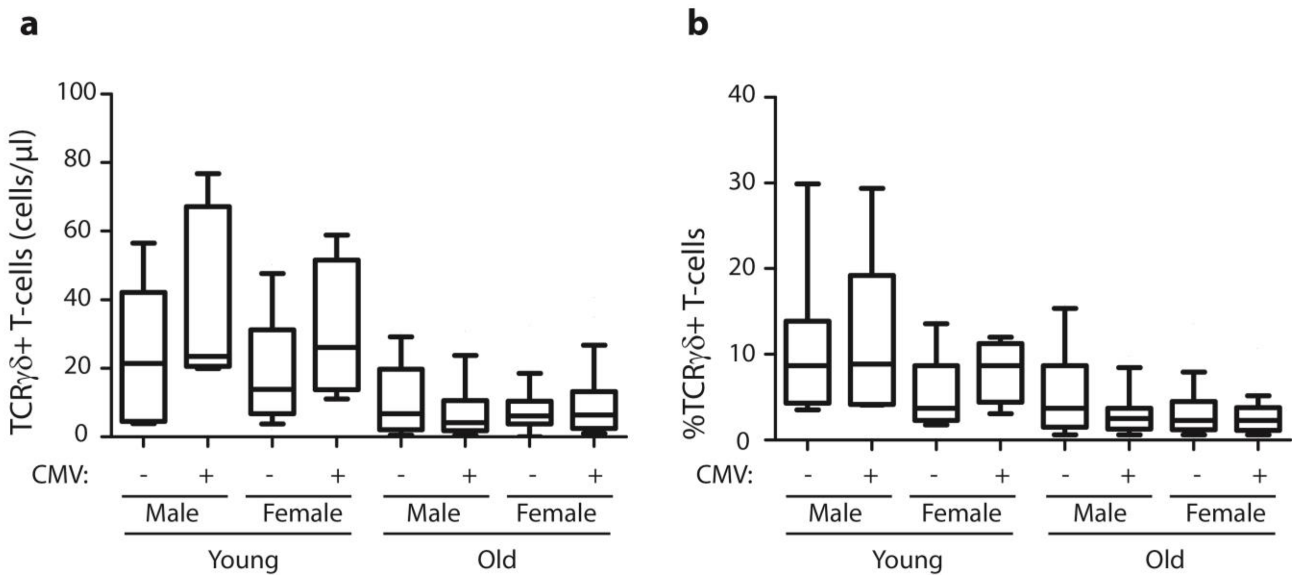

Figure S4. Effect of CMV, gender and age on V-gene usage. Supplementary to Figure 4 and Supplementary Table 4. **(a)** Absolute numbers and **(b)** percentages of total TCRγδ+ T-cells in young / elderly CMV- and CMV+ males and females depicted in 10-90% box-whiskers-plots. Significance was tested by a Kruskal-Wallis test, followed by a post-Dunn's test. Significance of the Dunn's test is indicated in the plots: *, $p<0.05$; **, $p<0.01$; ***, $p<0.001$.

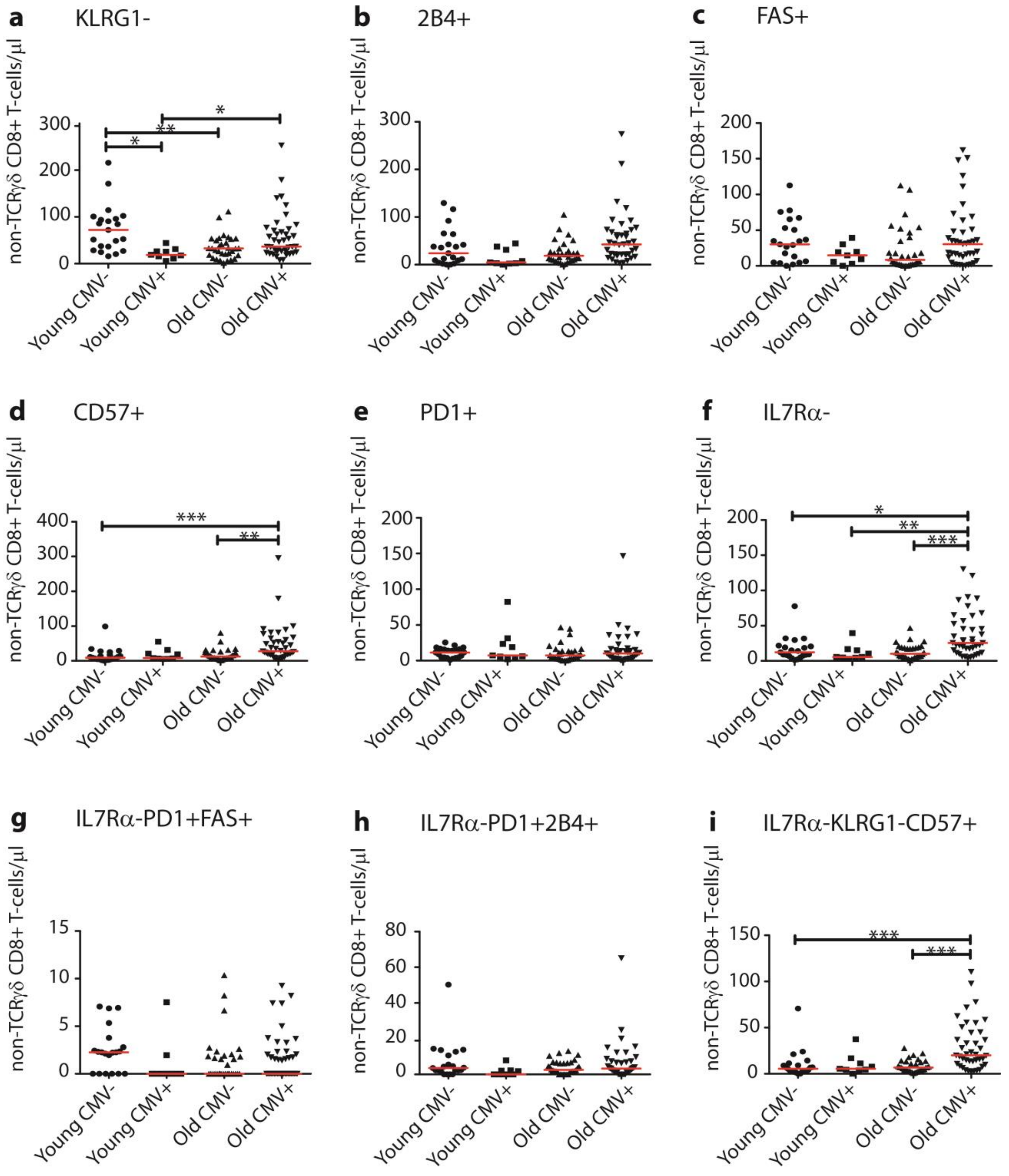

Figure S5. Effect of CMV on the exhaustion and senescence of nonTCRγδ (TCRαβ+) CD8+ T-cells.

Absolute numbers of TCRαβ+ (TCRγδ-negative T-cells) CD8+ T-cells **(a)** lacking KLRG1, **(b)** expressing 2B4, **(c)** FAS death receptor, **(d)** CD57, **(e)** PD1, and **(f)** lacking IL7Rα. Absolute numbers of TCRαβ+CD8+

IL7Rα- T-cells coexpressing **(g)** PD1 and FAS, **(h)** PD1 and 2B4, **(i)** CD57 and lacking KLRG1. Scatter plots are indicated with the median. Significance was tested by a Kruskal-Wallis test, followed by a post-Dunn's test. Significance of the Dunn's test is indicated in the plots: *, p<0.05; **, p<0.01; ***, p<0.001.

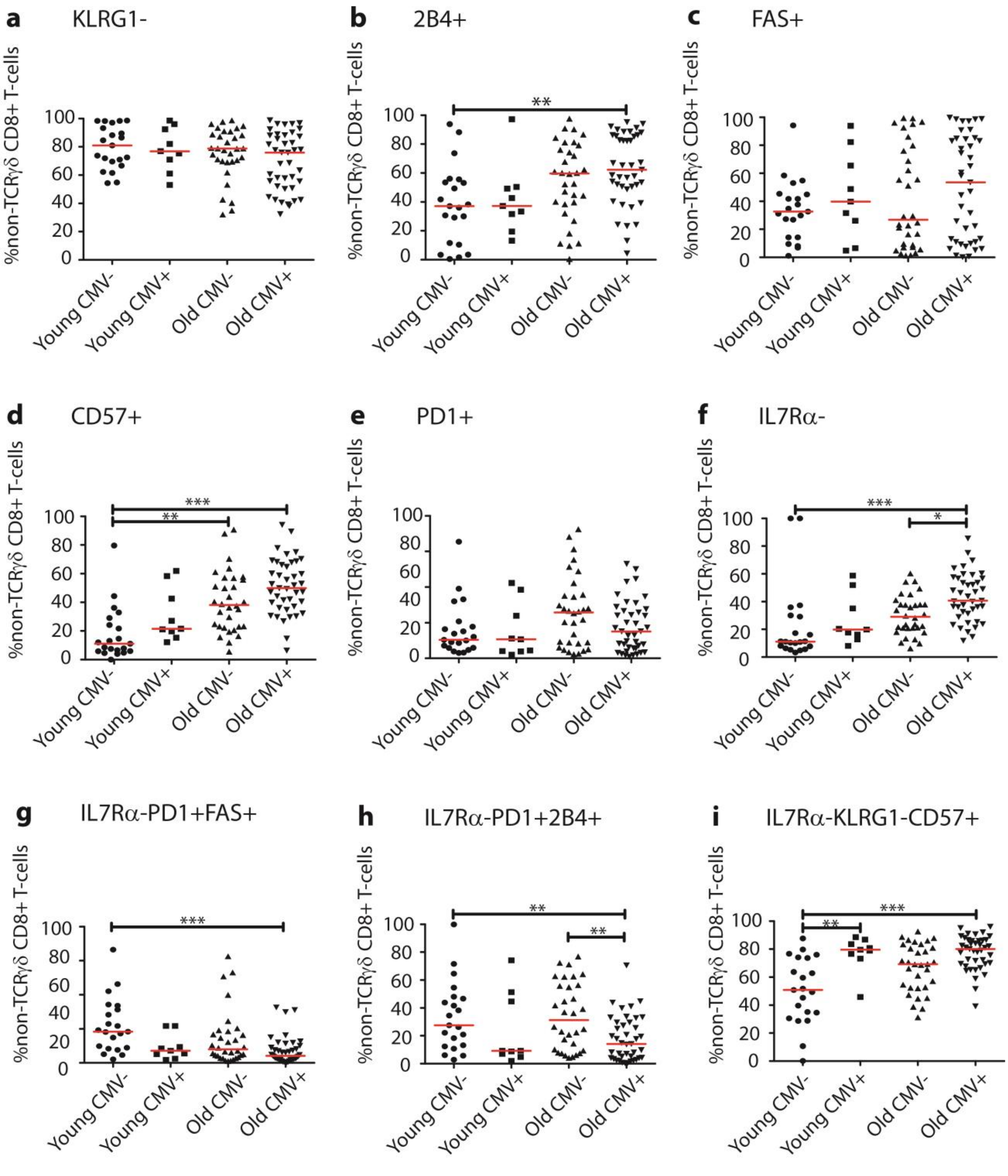

Figure S6. Relative effects of CMV on the exhaustion marker profile of nonTCRγδ (TCRαβ+) CD8+ T-cells. (a) Percentages of KLRG1-, **(b)** 2B4+, **(c)** FAS+, **(d)** CD57+, **(e)** PD1+ and **(f)** IL7Rα- TCRαβ+ (TCRγδ-

negative T-cells) CD8+ T-cells are depicted in 10-90% box-whiskers-plots. **(g)** Percentages of IL7Rα-TCRαβ+ T-cells co-expressing PD1 and FAS, **(h)** PD1 and 2B4 and **(i)** lacking KLRG1 with expression of CD57 depicted in scatterplots indicated with the medians. Significance was tested by a Kruskal-Wallis test, followed by a post-Dunn's test. Significance of the Dunn's test is indicated in the plots: *, $p<0.05$; **, $p<0.01$; ***, $p<0.001$.

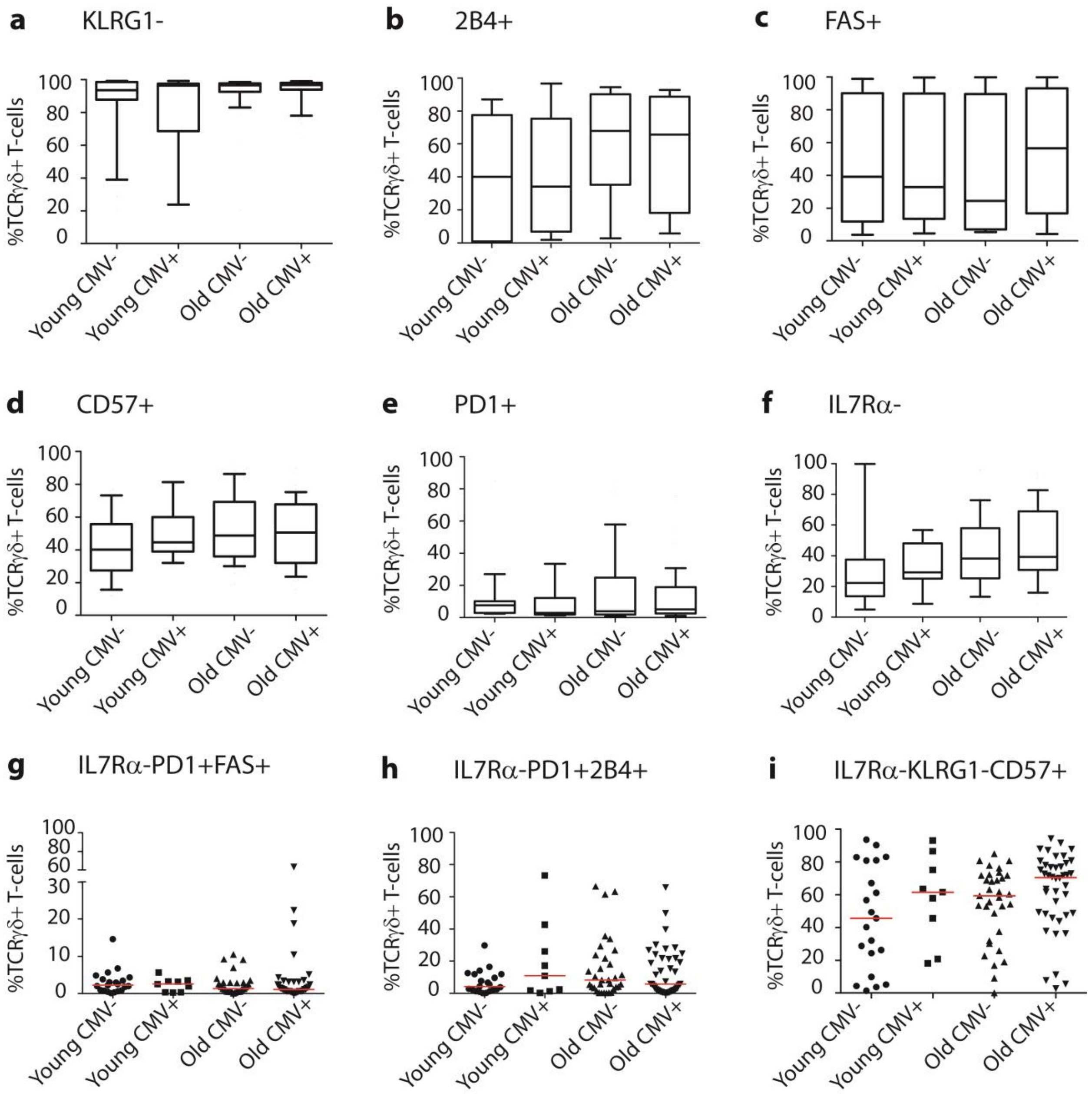

Figure S7. Relative effects of CMV on the exhaustion marker profile of TCRγδ+ T-cells. (a) Percentages of KLRG1-, **(b)** 2B4+, **(c)** FAS+, **(d)** CD57+, **(e)** PD1+ and **(f)** IL7Rα- TCRγδ+ T-cells are depicted in 10-90% box-whiskers-plots. **(g)** Percentages of IL7Rα- TCRγδ+ T-cells co-expressing PD1 and FAS, **(h)** PD1 and

2B4 and **(i)** lacking KLRG1 with expression of CD57 depicted in scatterplots indicated with the medians.

Significance was tested by a Kruskal-Wallis test, followed by a post-Dunn's test.

Supplementary Tables

Supplementary Table 1. Antibody details

Tube		Fluorochrome									
		**BV421**	**BV510**	**BV605**	**FITC**	**PerCP-Cy5.5**	**PE**	**PE-CF594**	**PE-Cy7**	**APC**	**APC-H7**
**1**	**Antibody**	**CD27**	**CD4**	**CD45RA**	**TCRVδ1**	**TCRVδ2**	**TCRVγ9**	**CD3**	**TCRαβ**	**CD25**	**CD8**
	Clone	O323	OKT4	HI100	TS8.2	B6	B3.1	UCHT1	IP26	2A3	SK1
	Manufacturer	BioLegend	BioLegend	BioLegend	Thermo Scientific	BioLegend	BD Biosciences	BD Biosciences	BioLegend	BD Biosciences	BD Biosciences
**2**	**Antibody**	**CD27**	**CD4**	**CD45RA**	**CD45RO**		**CD197**	**CD3**	**TCRγδ**	**CD28**	**CD8**
	Clone	O323	OKT4	HI100	UCHL1		3D13	UCHT1	11F2	CD28.2	SK1
	Manufacturer	BioLegend	BioLegend	BioLegend	DAKO		eBiosciences	BD Biosciences	BD Biosciences	BD Biosciences	BD Biosciences
**3**	**Antibody**	**CD127**	**CD4**	**CD95**	**KLRG1**	**CD244**	**CD279**	**CD3**	**TCRγδ**	**CD57**	**CD8**
	Clone	TU27	OKT4	DX2	2F1/KLRG1	C1.7	MIH4	UCHT1	11F2	NK-1	SK1
	Manufacturer	BioLegend	BioLegend	BioLegend	BioLegend	BioLegend	BD Biosciences	BD Biosciences	BD Biosciences	BD Biosciences	BD Biosciences
